# Are mouse models of asthma appropriate for investigating the pathogenesis of airway hyper-responsiveness?

**DOI:** 10.3389/fphys.2012.00312

**Published:** 2012-07-31

**Authors:** Rakesh K. Kumar, Paul S. Foster

**Affiliations:** ^1^Department of Pathology, School of Medical Sciences, University of New South WalesSydney, NSW, Australia; ^2^Discipline of Microbiology and Immunology, School of Biomedical Sciences and Pharmacy, University of Newcastle and Hunter Medical Research InstituteNewcastle, NSW, Australia

**Keywords:** airway hyper-responsiveness, airway inflammation, airway remodeling, animal models, asthma

## Abstract

Whether mouse models of chronic asthma can be used to investigate the relationship between airway inflammation/remodeling and airway hyper-responsiveness (AHR) is a vexed question. It raises issues about the extent to which such models replicate key features of the human disease. Here, we review some of the characteristic pathological features of human asthma and their relationship to AHR and examine some limitations of mouse models that are commonly used to investigate these relationships. We compare these conventional models with our mouse model of chronic asthma involving long-term low-level inhalational challenge and review studies of the relationship between inflammation/remodeling and AHR in this model and its derivatives, including models of an acute exacerbation of chronic asthma and of the induction phase of childhood asthma. We conclude that while extrapolating from studies in mouse models to AHR in humans requires cautious interpretation, such experimental work can provide significant insights into the pathogenesis of airway responsiveness and its molecular and cellular regulation.

## Introduction

Mouse models *can* certainly be employed to investigate the relationship between asthmatic inflammation/airway remodeling and airway hyper-responsiveness (AHR): they have been and continue to be used for such research. Whether they *should* be, i.e., whether the models are sufficiently similar to the human disease to be suitable for the purpose, is a separate question altogether. It raises, in turn, questions about the extent to which such models replicate key features of the human disease and about what exactly is being measured when AHR is assessed in animals. This review focuses on the first of those considerations: technical issues related to the measurement of AHR in mouse models and their relevance to physiological assessment in humans are examined elsewhere in this issue.

Whether mouse models replicate the features of human asthma with sufficient fidelity is a critically important question, which needs to be addressed both by experimentalists and by journal reviewers. Of course all experimental models have limitations, but if the model being investigated is not appropriate to the outcomes being assessed, it may be difficult to extrapolate from the experimental data to the human disease. While this has led some to suggest that mouse models of asthma have yielded little that has been able to be translated into improvements in therapy (Persson, 2002; Boyce and Austen, 2005; Wenzel and Holgate, 2006), many of the carefully performed studies in mouse models have yielded much interesting information about pathogenetic mechanisms of asthma.

To advance discussion about the relevance of mouse models, it would be appropriate to briefly review the characteristic pathological features of human asthma and their relationship to AHR. Some limitations of commonly used mouse models can then be examined, and the merits of a potential approach to overcoming these limitations can be assessed. In drawing comparisons, it is worth emphasizing that available mouse models are largely relevant only to allergic/eosinophilic asthma in humans. The overwhelming majority of children with asthma have evidence of allergy/atopy, but asthma of adult onset is frequently non-eosinophilic (Kumar and Jeffery, 2012). The pathological features described below relate to human allergic asthma.

## Airway inflammation in asthma

Airway inflammation is a characteristic feature of human asthma. The pathological changes are in general restricted to the conducting airways, mostly involving major bronchi, but also smaller airways in severe disease (James, 2009). Alveolar inflammation has been described in some settings (Kraft et al., 1996; De Magalhaes Simoes et al., 2005), but it should be emphasized that asthma is not primarily a condition involving the lung parenchyma.

**Figure 1:**
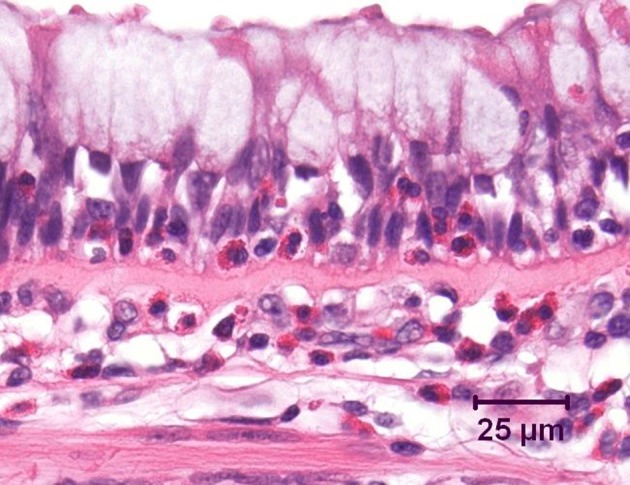
**Bronchus from an asthmatic patient, demonstrating numerous eosinophils, both within the airway epithelium and in the underlying lamina propria.** Note the marked goblet cell change in the epithelium and the prominent zone of subepithelial fibrosis. Hematoxylin and eosin.

Even in the earliest stages of mild clinical asthma, there is accumulation of chronic inflammatory cells in the mucosa of the larger conducting airways (Holgate et al., 1992; Laitinen et al., 1993). These include increased numbers of lymphocytes, plasma cells, mast cells, and macrophages. As has long been emphasized, in allergic asthma the majority of lymphocytes within the airway wall are CD4^+^ cells which exhibit a T helper type 2 (Th2) profile of cytokine secretion, characterized by production of interleukin (IL)-4, IL-5, and IL-13 (Hamid et al., 1991; Yung et al., 1995). Lymphocytes and mast cells are also prominent deeper in the airway wall, including in the smooth muscle layer (Brightling et al., 2002; Begueret et al., 2007). Superimposed upon this background is an acute inflammatory component, which is typically associated with recruitment of eosinophils into the mucosa (Bradley et al., 1991; Shaver et al., 1997) and in particular into the epithelial layer (Figure 1). Indeed, intraepithelial eosinophils are considered to be a distinctive feature of asthmatic inflammation (Bousquet et al., 1990), whereas recruitment of neutrophils into the airways is uncommon in mild to moderate asthma.

In patients with asthma, there has long been controversy about whether airway inflammation correlates with AHR (Sont et al., 1996; Crimi et al., 1998). This may in part be a consequence of the use of different stimuli to assess airway responses. AHR to indirect stimuli, such as distilled water, hypertonic saline, or mannitol, seems to correlate more closely with airway inflammation than does the response to bronchoconstrictor agents (Chetta et al., 1996; Joos et al., 2003).

## Investigating the relationship between inflammation and AHR in mouse models

**Figure 2:**
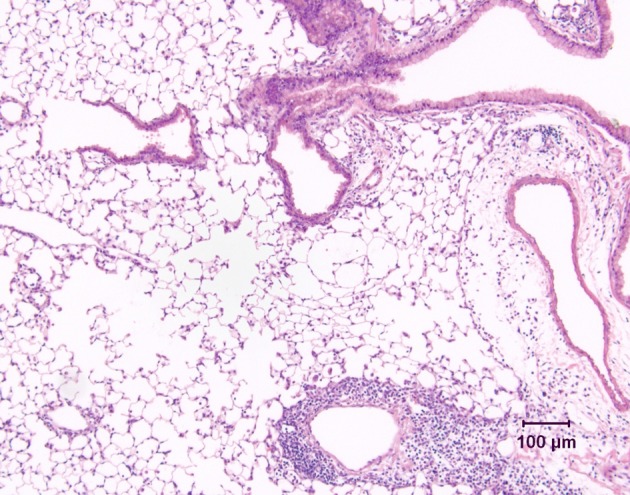
**Lung tissue demonstrating the inflammatory response in a conventional mouse model of allergic airway inflammation.** Following short-term, uncontrolled inhalational challenge of a sensitized animal with aerosolized ovalbumin, there is marked perivascular inflammation (below, right) and edema (right) but relatively limited peribronchiolar inflammation. Hematoxylin and eosin.

Most mouse models of asthma involve short-term challenge of sensitized mice with antigen delivered either by inhalational exposure that is uncontrolled (i.e., with no attempt made to limit the mass concentration of aerosol to which the animals are exposed and no monitoring of the concentration of aerosol in the breathing zone of the animals) or intratracheally. These models are experimentally convenient, but given the nature of the antigenic challenge, they are primarily models of severe acute allergic inflammation. Thus they are very useful for investigating the basis of allergic inflammation, including the role of CD4^+^ Th2 cells, as well as the various cellular and molecular pathways involved in regulating their activity. However, there are important differences in the pattern of airway inflammation compared to human asthmatics. Notably, inflammation is by no means limited to the conducting airways, but is usually associated with accumulation of large numbers of perivascular inflammatory cells, as well as with marked edema around blood vessels (Figure 2). The severity and extent of peribronchiolar inflammation are variable. There is also a relative over-representation of eosinophils, which comprise 40–80% of the cells in the bronchoalveolar lavage fluid (BALF) of these mice, in contrast to the 1–5% eosinophils in BALF from human asthmatics.

Importantly, chronic inflammation of the airway wall is conspicuously absent in these models, which means that they cannot be used for investigation of the relationship between chronic airway inflammation and AHR. Thus their relevance is likely to be limited to AHR in severe asthma or exacerbations of asthma, subject of course to the problems associated with the lack of comparability of techniques used to assess AHR in mice as compared to humans. There are also questions about the relative contribution of changes in large *vs*. small airways to the AHR that is able to be demonstrated. *Ex vivo* studies in these mouse models have provided evidence of altered contractility of bronchial smooth muscle in response to allergen or a non-specific bronchoconstrictor agent (Chiba et al., 2005; Chew et al., 2008) and a component of AHR, measured using the most appropriate available techniques, undoubtedly does originate from the conducting airways. However, it has been suggested that in short-term models, AHR may at least in part be explained on the basis of thickening of the airway mucosa and an increased propensity of small airways to close, without any increase in the degree of airway smooth muscle shortening (Wagers et al., 2004; Lundblad et al., 2007). Notwithstanding this caveat, airway closure in response to a bronchoconstrictor challenge is also a contributory mechanism of AHR in human asthmatics (Berend et al., 2008).

It is also important to realize that the information which may be gained from the use of mouse models to investigate development of AHR is influenced by key experimental variables such as the time point of examination after allergen challenge. For example, we have shown that prolonged AHR (i.e., altered responsiveness detectable at 1 week after allergen challenge, demonstrable as primarily originating from the conducting airways) may largely be driven by an interaction between interferon-γ and pulmonary macrophages rather than involving eosinophilic inflammation (Yang et al., 2010).

## Airway wall remodeling in asthma

Pathological remodeling in asthma refers to structural changes in the airway wall. Early on, even in mild or moderate asthma, there is prominent thickening of a fibrillary layer subjacent to the epithelium, which is highly characteristic of the disease (Jeffery, 2001). This is primarily associated with deposition of type III and type V collagens, together with other matrix proteins including laminins (Altraja et al., 1996), tenascin (Laitinen et al., 1997), periostin (Takayama et al., 2006), and a variety of proteoglycans. It is variously referred to as subepithelial fibrosis or thickening of the reticular basement membrane (RBM) and is associated with evidence of Th2-driven allergic inflammation (Woodruff et al., 2009) and the presence of increased numbers of subepithelial myofibroblasts (Brewster et al., 1990). At least in large airways, RBM thickness correlates well with overall thickening of airway wall, so that assessment of the thickness of the subepithelial layer in bronchial biopsies may be a useful indicator of airway remodeling (James et al., 2002). However, its relevance to functional changes in asthma is unclear, and recent investigations suggest that subepithelial thickening may be a consequence of bronchoconstriction rather than a contributor to airflow obstruction (Grainge et al., 2011).

Perhaps the most important feature of asthmatic airway remodeling is thickening of the airway smooth muscle layer (James and Carroll, 2000; James et al., 2012). This includes hypertrophy and hyperplasia of the muscle cells, as well as altered deposition of extracellular matrix (Woodruff et al., 2004; Begueret et al., 2007; Pini et al., 2007; Hassan et al., 2010). Increased smooth muscle mass is highly likely to contribute to AHR because thickening of the airway wall may decrease the degree of airway smooth muscle shortening required to occlude the lumen (James et al., 1989; James and Wenzel, 2007).

Airway mucosal vascularity, especially increased density of newly formed small vessels, is also a feature of remodeling in mild to moderate asthma (Li and Wilson, 1997; Salvato, 2001; Feltis et al., 2006). These vessels may have increased permeability and thus contribute to airway wall edema leading to airflow obstruction (Khor et al., 2009), although the relevance of this to AHR is unknown.

Another characteristic feature of remodeling in human asthmatics is an increase in the number of mucin-secreting goblet cells in the surface epithelium, due to both hyperplasia and metaplasia (the latter representing altered differentiation and seen mostly in small airways; Ordonez et al., 2001). Again, the relationship between these changes and AHR in patients has not been defined, further emphasizing that the relationship between airway remodeling and AHR in asthmatics remains poorly understood.

## Investigating the relationship between remodeling and AHR in mouse models

As noted above, most commonly used mouse models involve short-term challenge of sensitized animals with large amounts of antigen, triggering severe acute allergic inflammation. Unsurprisingly, such models replicate few features of airway remodeling, although mucous cell change is a very rapidly developing response (Ohkawara et al., 1997) and some studies have yielded interesting information about its potential role in AHR (Nakanishi et al., 2001; Long et al., 2006). In the past dozen years, a number of investigators have sought to develop better animal models based on extending the period of antigenic challenge to 4–8 weeks (Braun et al., 1999; Hogaboam et al., 2000; Tanaka et al., 2001; Henderson et al., 2002; Jain et al., 2002; Leigh et al., 2002; Kenyon et al., 2003; Johnson et al., 2004; Pitchford et al., 2004; Yang et al., 2004; McMillan et al., 2005; Wegmann et al., 2005; Locke et al., 2007; Shinagawa et al., 2007). Many of these do exhibit various features of airway wall remodeling: most reports describe marked goblet cell change and several also demonstrate evidence of increased airway smooth muscle. A single study reported increased airway vascularity in such a model (Su et al., 2008).

However, almost all of these chronic challenge models still have shortcomings. Notably, because of the unregulated exposure to high mass concentrations of inhaled antigen, repeated challenge is frequently associated with severe pulmonary inflammation, sometimes with formation of multinucleated giant cells and even granulomas (Mathew et al., 2001). This response appears to be somewhat variable and possibly dependent on the strain of mice used, because several reports suggest that there may instead be progressive downregulation of airway inflammation and Th2 responses (Irifune et al., 1998; Yiamouyiannis et al., 1999; Sakai et al., 2001; Leigh et al., 2002; Pitchford et al., 2004). Furthermore, the marked peribronchiolar accumulation of collagen that is observed in such models is quite distinct from the limited zone of subepithelial thickening in conducting airways that is characteristic of human asthma. This peribronchiolar scarring, which often extends further including around blood vessels, is not synonymous with subepithelial fibrosis, despite the widespread misuse of the term by investigators, and should instead be described as airway wall fibrosis. Although small airway remodeling has been demonstrated in severe asthmatics (Dolhnikoff et al., 2009), airway fibrosis is not a characteristic lesion of human asthma. Clearly, it would be unwise to draw conclusions about the relationship between airway remodeling and AHR in such a setting.

## A more realistic mouse model of mild chronic asthma

We have described a mouse model of chronic asthma that involves long-term challenge of sensitized mice with carefully controlled low mass concentrations of aerosolized ovalbumin (approximately 3 mg/m^3^ which is some 100–1000 times lower than used in conventional models; Temelkovski et al., 1998). This exhibits changes of chronic asthma that closely resemble the human disease, both in terms of pattern and spatial distribution of cellular responses. Airway disease is established by 4 weeks of inhalational challenge (30 min/day, 3 days/week) with lesions including eosinophil recruitment into the epithelial layer of the conducting airways, chronic inflammation in the airway wall with accumulation of T-lymphocytes and plasma cells, and changes of remodeling such as sub-epithelial fibrosis, epithelial hypertrophy, and goblet cell metaplasia. Importantly, there is minimal parenchymal inflammation, i.e., no perivascular or peribronchiolar inflammation in the lungs (Temelkovski et al., 1998). In terms of pathogenesis, in this model there is clearly a role for both Th2 and Th1 cytokines, as demonstrated in studies using gene-targeted animals and neutralizing antibodies (Kumar et al., 2002, 2004a,c). This model has, therefore, been widely acknowledged to represent a significant improvement in terms of the fidelity with which it reproduces features of human asthma (Shore, 2003; Fulkerson et al., 2005; Nials and Uddin, 2008).

Can this model be used to investigate the pathogenesis of AHR and, in particular, its relationship to airway remodeling? Our studies suggest that, at the very least, the cytokine mechanisms involved in development of AHR following low-level chronic challenge may be different to those demonstrated in short-term models assessed at early time points after challenge, because there appears to be less dependency on Th2 responses and a significant role for interferon-γ (Kumar et al., 2004c). Moreover, although the presence of airway remodeling is often correlated with evidence of AHR, we have clearly demonstrated that these may be dissociated (Foster et al., 2000). We would not suggest that this model provides definitive answers, as we have previously identified some important limitations. Unlike chronic exposure models in which antigenic challenge is uncontrolled, we find no evidence of significant hypertrophy or hyperplasia of airway smooth muscle (Kumar et al., 2004b), which limits comparability to the human disease. In addition, as in other models in mice, there is no evidence of accumulation of mast cells in the airways (Kumar et al., 2000) so the potential contribution of these cells cannot be properly assessed.

## Modeling other aspects of asthma

Using our chronic challenge model as a substrate, we have also established a model of an allergen-induced acute exacerbation of chronic asthma, in which following low-level challenge for 4 weeks, animals were exposed for a 30-min period to a single challenge with 30 mg/m^3^ of aerosolized antigen (i.e., 10-fold higher than usual). This moderate-level challenge is associated with more marked airway inflammation, which extends further distally than in the chronic challenge model (Siegle et al., 2006). Compared to short-term uncontrolled challenge models, however, the inflammatory response is relatively mild and the numbers of eosinophils, lymphocytes and neutrophils in BALF are modest. Table 1 compares some key features of the human disease with short-term, high-level challenge models and the models that we have developed.

**Table 1:** **Comparison of human asthma and various mouse models**.

	**Chronic human asthma**	Mouse model of chronic asthma^*^	**Short-term mouse models**	**Human acute exacerbation**	Mouse model of acute exacerbation^*^
Eosinophils in BALF	+	±	++++	++	+
Lymphocytes in BALF	+	+	+	++	++
Neutrophils in BALF	−	−	++	++	++
Chronic inflammation in tissue	+	+	−	+	+
Airway wall remodeling	+	+	−	+	+
Distal airway inflammation	−	−	+++	+	+
AHR originating from distal airways	−	−	++	+	+

* These columns refer to the models that we have developed, as described in the text.

Does our model permit investigation of the pathogenesis of AHR in patients with an acute exacerbation of chronic asthma? Quite possibly, because these animals demonstrate a pattern of AHR distinct from that seen in the chronic challenge model, presumably reflecting the distal airway involvement (Siegle et al., 2006). Development of AHR appears not to be dependent on eosinophil recruitment and is relatively resistant to treatment with glucocorticosteroids (Ito et al., 2008).

More recently, we have extended the approach of using controlled inhalational challenge to studies seeking to model the induction phase of asthma in childhood, with sensitization early in life via the respiratory tract. Because inhalational sensitization/challenge with an innocuous protein antigen such as ovalbumin does not elicit an allergic immunological response and indeed may lead to tolerance (Gerhold et al., 2003; Wang and McCusker, 2006), early life airway epithelial injury by environmental factors, which promotes sensitization, appears to be essential for the development of features of asthma. We have demonstrated a crucial interaction between neonatal respiratory viral infection and respiratory sensitization/challenge in the pathogenesis of an asthmatic phenotype (Siegle et al., 2010) and have subsequently also investigated the role of early life exposure to environmental particulates in progression to an asthmatic pattern of inflammation (Herbert et al., submitted for publication). These studies have highlighted the likely importance of persistent epithelial injury in the development of AHR in such models.

## The bottom line

A reasonable answer to the question “Can mouse models be used to investigate the relationship between airway inflammation/remodeling and AHR?” is: up to a point, but it is important to recognize the associated problems and limitations. Both researchers and referees need to be alert to such issues. As an example, smooth muscle changes that appear to be important in the development of AHR in human asthmatics are not reproduced at all well in mouse models except when associated with numerous confounding lesions, which are often ignored. Similarly, the literature contains many reports of studies in which severe peribronchiolar fibrosis is used as an index of remodeling and is then correlated with effects on AHR, yet such fibrosis is not a feature of the airways in patients with asthma. A lack of appreciation of the pathological changes in human asthma can lead to the use of suboptimal animal models and avoidable weaknesses in experimental design.

Given the complexity of asthma, however, it will always be the case that no single animal model—in mice or any other species—will be a perfect system for studies of the pathogenesis of AHR or any other aspect of the human disease. For experimentalists to be able to design/select mouse models that are appropriate for the investigation of the question of interest, this complexity needs to be unraveled. Experimental work focusing on Th2-mediated responses was driven by the recognition that this was a key feature of inflammation in the airways of human asthmatics. However, current approaches to stratifying inflammatory phenotypes of asthma in patients (e.g., based on examination of induced sputum) cannot readily be translated to mouse models. In turn, this means that translation of experimental findings back to the appropriate subset of patients is difficult. Phenotyping of asthma based on pathological changes at the cellular and molecular level, rather than on clinical manifestations and assessment of pulmonary function, is likely to facilitate the design of better studies using appropriate mouse models that shed light on pathogenesis, which in turn can lead to the development of worthwhile therapeutic interventions.

### Conflict of interest statement

The authors declare that the research was conducted in the absence of any commercial or financial relationships that could be construed as a potential conflict of interest.
